# MGcV: the microbial genomic context viewer for comparative genome analysis

**DOI:** 10.1186/1471-2164-14-209

**Published:** 2013-04-01

**Authors:** Lex Overmars, Robert Kerkhoven, Roland J Siezen, Christof Francke

**Affiliations:** 1 Centre for Molecular and Biomolecular Informatics, Radboud University Nijmegen Medical Centre, Geert Grooteplein Zuid 26-28, Nijmegen, 6525GA, The Netherlands; 2 TI Food and Nutrition, P.O. Box 557, Wageningen, 6700AN, The Netherlands; 3 Netherlands Bioinformatics Centre, P.O. Box 9101, Nijmegen, 6500HB, The Netherlands; 4 Microbial Bioinformatics, Eikelakkers 2, Ede, 6711TE, The Netherlands; 5 CFLSc, Prinsenhof 12, Eindhoven, 5616TE, The Netherlands

**Keywords:** Comparative genomics, Function annotation, Genome visualization, Network reconstruction, Regulatory element, Transcription regulation

## Abstract

**Background:**

Conserved gene context is used in many types of comparative genome analyses. It is used to provide leads on gene function, to guide the discovery of regulatory sequences, but also to aid in the reconstruction of metabolic networks. We present the Microbial Genomic context Viewer (MGcV), an interactive, web-based application tailored to strengthen the practice of manual comparative genome context analysis for bacteria.

**Results:**

MGcV is a versatile, easy-to-use tool that renders a visualization of the genomic context of any set of selected genes, genes within a phylogenetic tree, genomic segments, or regulatory elements. It is tailored to facilitate laborious tasks such as the interactive annotation of gene function, the discovery of regulatory elements, or the sequence-based reconstruction of gene regulatory networks. We illustrate that MGcV can be used in gene function annotation by visually integrating information on prokaryotic genes, like their annotation as available from NCBI with other annotation data such as Pfam domains, sub-cellular location predictions and gene-sequence characteristics such as GC content. We also illustrate the usefulness of the interactive features that allow the graphical selection of genes to facilitate data gathering (e.g. upstream regions, ID’s or annotation), in the analysis and reconstruction of transcription regulation. Moreover, putative regulatory elements and their corresponding scores or data from RNA-seq and microarray experiments can be uploaded, visualized and interpreted in (ranked-) comparative context maps. The ranked maps allow the interpretation of predicted regulatory elements and experimental data in light of each other.

**Conclusion:**

MGcV advances the manual comparative analysis of genes and regulatory elements by providing fast and flexible integration of gene related data combined with straightforward data retrieval. MGcV is available at http://mgcv.cmbi.ru.nl.

## Background

The number of sequenced prokaryotic genomes keeps expanding at a rapid pace. As a result, much of the function annotation of genes and other sequence elements relies increasingly on automated pipelines. Despite this tendency, human interference remains indispensable to translate genomic data correctly to biological meaning. Gene context and its evolutionary conservation is one of the genomic properties that can greatly aid the related (manual) genome analyses. The gene context provides many clues concerning function and biological role of a gene in a prokaryote [1,2]. Gene context data thus benefits the reconstruction of the metabolic network [3-5]. Moreover, conserved gene context can also be applied to guide the identification of regulatory elements and therewith the reconstruction of the transcription regulatory network (e.g. [6-9]).

From a practical point of view, a comprehensive visualization of genomics data and information on function facilitates the process of data integration, and thereby reduces the time needed for interpretation. There are several ways to achieve this goal, as reflected by the variety in genome browsers and annotation platforms that have been developed. Conventional genome browsers include for instance UCSC genome browser [10], Artemis [11] and GBrowse [12]. This type of genome browser is characterized by a generic, highly configurable setup (i.e. typically, users can upload their genomes in genbank- and/or gff3-format) and display genomic data in separate ‘tracks’. On the other hand, resources such as IMG [13], Microscope [14], MicrobesOnline [15] and the SEED [16] serve as annotation platforms by providing the user genomic data, analysis tools and visualization options. In 2004 we introduced the Microbial Genome Viewer [17]. This web-based genome viewer allowed users to explore bacterial genomes in linear maps and create a genome-wide visualization of data in circular maps. Yet, other tools have a more specific focus. For instance, BAGET allows users to retrieve the gene-context for a single gene [18], whereas GeConT 2 allows users to visualize the genomic context of query genes [19]. Some tools specifically address conservation of gene order between orthologous genes, also denoted as “synteny”. For instance, GeneclusterViz [20], GCView [21], PSAT [22] and Absynte [23] provide a local gene context comparison based on blast (−like) similarity searches.

In the public domain, various resources provide organism specific reconstructions of particular regulons through the integration of genome sequence data and stored motifs. Examples of these are PEPPER [24], RegulonDB [25], RegTransBase [26], PRODORIC [27], RegPrecise [28], ProdoNet [29], FITBAR [30], RegAnalyst [31] and MicrobesOnline [15]. Most of these resources enable automated predictions of regulatory sites based on stored motifs collected from literature. Some resources also in addition allow for *de novo* motif discovery, using tools such as MEME [32], Tmod [33] and GIMSAN [34], which were developed to identify significantly overrepresented sequence motifs.

The versatility of the above resources comes at the cost of some flexibility and speed. We have therefore developed the web-application MGcV, which aims specifically to serve as an integrative visual interface to speed up a manual genome analysis. MGcV is a light-weight and flexible viewer that provides: i) a comparative view of the genomic context for query genome segments, like genes, sets of genes, or (user defined-) gene trees; ii) the integration of information on gene function enriched with additional annotation data such Pfam domains and sub-cellular location-predictions within a single ‘track’; iii) the possibility to visually select genes and extract diverse gene-linked information, like upstream regions, protein sequence or function annotation; and iv) the possibility to upload and integrate experimental data and user-defined regulatory elements in adaptable views. MGcV thus enables the exploitation of gene context information in the annotation of gene function, the analysis of the evolutionary conservation of that context, the recovery of associated regulatory elements and the ranked comparative view of the identified elements in combination with microarray- or RNA-seq data. Hereby MGcV provides a visual heart to the manual sequence-based analysis of gene-function and gene-regulation in bacteria.

## Methods

### Data resources

The genome and protein sequences, the associated gene identifiers and function annotations (e.g. trivial names, COG categories, protein names) of all publicly available bacterial genomes are obtained from the FTP server of NCBI RefSeq [35,36]. Uniprot accessions mapped to NCBI GI-codes are retrieved from the Uniprot FTP server [37,38]. Pfam domains are obtained from the FTP server of EBI [39,40]. Gene-sequence characteristics like GC-content are calculated using in-house scripts. Sub-cellular location predictions are obtained from the PSORTdb website [41,42]. The data is updated on a weekly basis and stored in a local MySQL database to enable fast access. The microarray data that are used to illustrate the capabilities of MGcV in the second case study were taken from [43].

## Implementation

**Figure 1:**
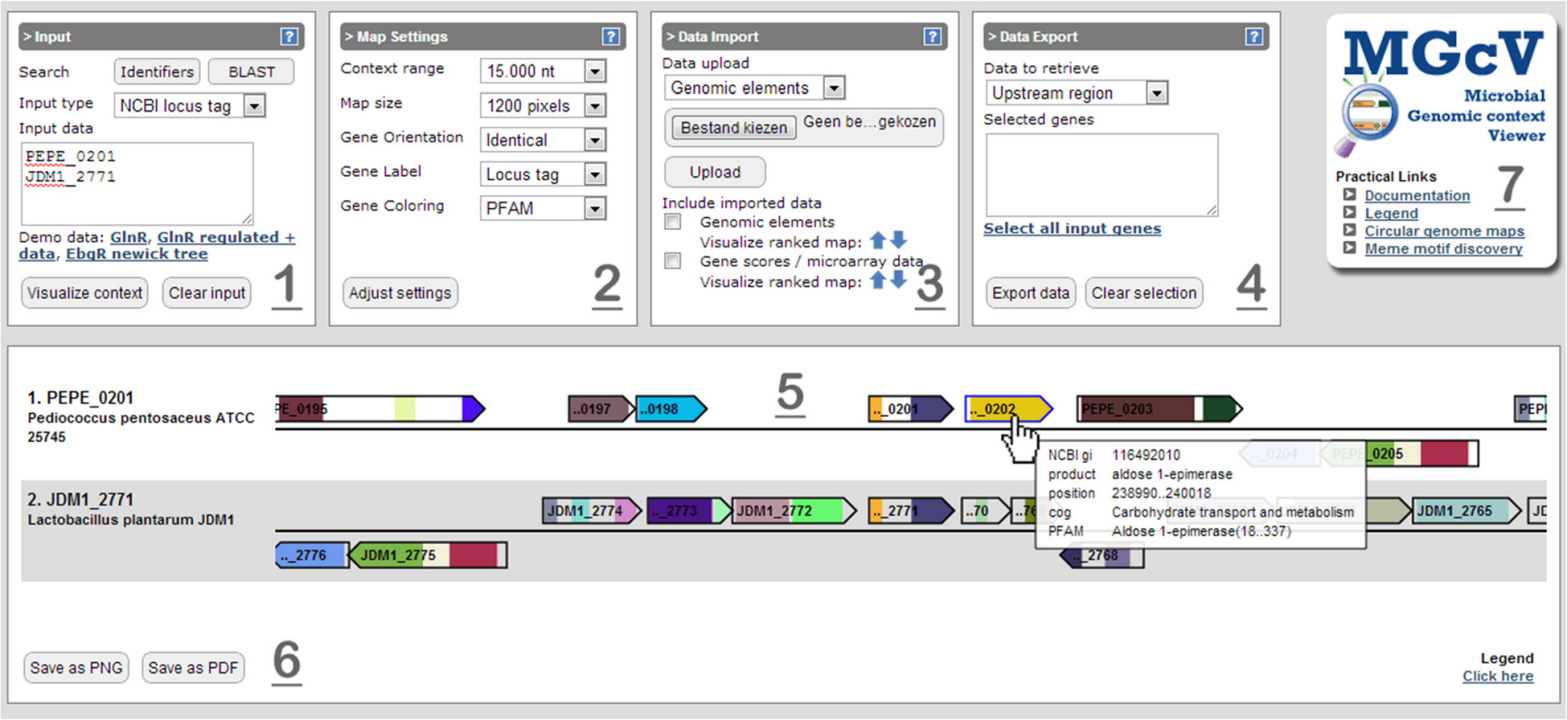
**The interface of MGcV.** The interface comprises seven separate modules. 1) Input: Copy/paste the input containing regions of interest and select the appropriate input-type. The context is visualized by clicking “Visualize context”. Input-types include lists of identifiers such NCBI GI-codes or NCBI locus tags, genomic regions, or phylogenetic trees in newick format. Identifiers can also be searched for using the buttons “Identifier” and “BLAST”. 2) In the map settings, the appearance of the comparative context map can be altered. Various settings can be changed, which include the scale, the orientation, the gene-label and the data on display (e.g. COG coloring, PFAM, protein location). To apply the changes the “Adjust settings”-button has to be clicked. 3) Data can be added via data import. Users can upload the position of genomic elements (e.g. regulatory elements) and gene-associated quantitative data (e.g. microarray data). Subsequently, the user can include the data in the map or generate a (ranked) comparative context map based on the uploaded data (blue arrows). 4) Sequence and functional data like upstream regions, gene sequences, cog categories, annotations can be obtained for visually selected genes via data export. Genes can be included or excluded by clicking. 5) The functional data related to a particular gene context is displayed in a single lane in the comparative genome map, thereby allowing for a direct comparison of the data between various strains or species. The maps are interactive; hovering the mouse over the map will display additional information (e.g. NCBI GI, gene product, COG) and clicking genes will tag them for data export. 6) The map can be converted to PNG- or PDF- format. 7) The interface is linked to a tutorial, to a color legend, to the circular viewer of the original MGV, and also directly to the MEME website.

MGcV is a web-application developed using a combination of python, javascript and SVG (Scalable Vector Graphics). We implemented MGcV as a single page application; the front-end makes server side calls through Jquery and AJAX and receives the response from the server. The interface consists of four boxes (see Figure 1). From left to right, these include: i) data input; ii) map settings; iii) data import and; iv) data export. The user can provide four types of input, NCBI RefSeq GI’s, NCBI locus tags, genomic positions (NCBI Refseq genome accession *tab* start *tab* stop), or a Newick-tree (the leaf labels must contain NCBI-GI code). In addition, the user can search for specific genes by providing for instance gene product or gene names or by performing a BLAST search. Query data supplied by the user is parsed and mapped to the corresponding gene context data using python scripts. Uploaded (phylogenetic) trees are processed with Newick utilities [44], which is also used to create a visual representation of the tree in SVG format. For the COG (NCBI) and Pfam annotations in the gene context maps a color scheme was assigned by designating a unique color to each identifier. In a similar way colors were assigned to the different protein location predictions. The applied color schemes can be found in the legend. Gene-associated quantitative data (e.g. GC-content) are converted in a red-to-green gradient which is projected on top of the genes. Likewise, gene-associated quantitative data uploaded by the user (e.g. microarray- or RNAseq- data) are converted in a red-to-green gradient. These data are then projected in a horizontal bar below the genes to allow the visual integration with annotation data and regulatory element predictions. Generated maps can be downloaded in SVG, PNG or PDF format. The conversion of SVG to PNG and PDF is done using “Batik Rasterizer” [45]. The interface and interactive maps allow the user to interact with the data. Map interactivity is achieved by ECMAscript; linked information on genes and other sequence elements can be inspected by mouse-over, whereas a mouse-click can be used to select genes for subsequent analysis and data retrieval. MGcV is operable in modern browsers like Firefox, Chrome and Internet Explorer, where for all browsers the latest version is recommended.

## Results

### Interface and functionality

#### Function annotation

The appropriate annotation of encoded function is essential for the correct interpretation of genomics data. The annotation process is initiated by the selection of genes and/or regions of interest. The flexible set-up of MGcV allows to generate an initial comparative context map simply by uploading a single identifier or a list of identifiers, like derived from a BLAST search, suffices to generate an initial comparative context map in MGcV. The uploaded identifiers may include NCBI gi-codes (RefSeq [36]), NCBI locus tags or genomic locations (designated by a RefSeq genome accession and position). In case the user does not have a list of gene identifiers, genes and their corresponding identifiers can be obtained via the built-in gene-search (input-box option “Identifiers”). In addition, a BLAST search can be performed to find proteins similar to a given protein sequence. The BLAST hits can be selected and used as input for MGcV. We have also implemented the possibility to upload and visualize any (phylogenetic) gene tree. The combined view of gene phylogeny and the gene context allows a quick evaluation of the potential for similarity in molecular function and biological role between the selected genes. The labeling of the genes (i.e. by trivial name, by locus tag, or by NCBI GI-code), and similarly, the coloring of the genes (i.e. by COG category [46], by GC%, by sub cellular location [47] or by Pfam domain [40]) enhances the evaluation process. In addition, the genomic range of the maps can be altered and an identical orientation of the genes of interest can be enforced for purposes of presentation. The added value of MGcV in the manual function annotation is illustrated in more detail below (first case study).

#### Identification and comparison of regulatory elements

The starting point for a sequence-based reconstruction of transcription regulation is the identification of genes whose upstream region might contain a regulatory element, like a transcription factor (TF) binding site (e.g. [6,7,9,48]). We and others have shown that the identification of specific TF binding sites is particularly successful in the case of conserved gene context (e.g. [8,49,50]). We experienced that the ability to select upstream regions on basis of a visual representation of that context considerably speeds up the analysis and therefore have implemented this upstream region selection in MGcV. Moreover, we have added a “data import” option to allow the visualization of the predicted location of regulatory elements together with microarray or RNA-seq data. In this way, the location prediction of regulatory elements and the experimental data can be interpreted more easily in light of each other. In addition, the view can be ranked according to similarity score (for binding site predictions) or expression ratio (for microarray or RNA seq data). In fact, such a ranked view of expression data and gene context is also extremely useful in the interpretation of transcriptome experiments. The new features are illustrated below in the second case study.

#### Data export

An important aspect of data integration in comparative genome analyses is the combination of sequence and, sequence and function identifiers. Collecting these identifiers for a selected set of genes can be time-consuming, especially when the information linked to the genes found associated on the genome has to be included. We have added a “data export” option in MGcV to accommodate the rapid and comprehensive collection of gene-related data. The user can graphically select genes of interest by mouse-click, where the selected genes are highlighted and included in the “data export”-box. Subsequently, the data to be retrieved can be selected. These include for example upstream DNA sequences, protein sequences or function-related data like for instance: length, protein function, COG category or Pfam domains. The export option can be used without actually using the context view to, for instance, collect quickly the protein sequence or Uniprot accession codes for a set of gene IDs.

### Case studies that illustrate the practical application of MGcV in manual comparative genome analysis

The main difference between MGcV and other resources is that MGvC is aimed to provide a platform to visually integrate one’s own data (i.e. data generated externally using other tools or obtained through experimentation) with annotation data and practical export options that enable further (external) analysis. Other resources, like for instance MicrobesOnline [15], in principle aim to offer a platform that is inclusive, i.e. that includes both calculation and visualization. Below we describe the results of two different manual comparative genome analysis using MGcV. In these two examples we highlight the flexible functionality of MGcV by visualizing the gene context and the associated functional information for a set of homologs that are present in a phylogenetic tree and by the visual integration of microarray data and de novo predictions of putative binding sites.

#### Case study 1: beta-galactosidases and associated regulators in Lactobacillus plantarum

**Figure 2:**
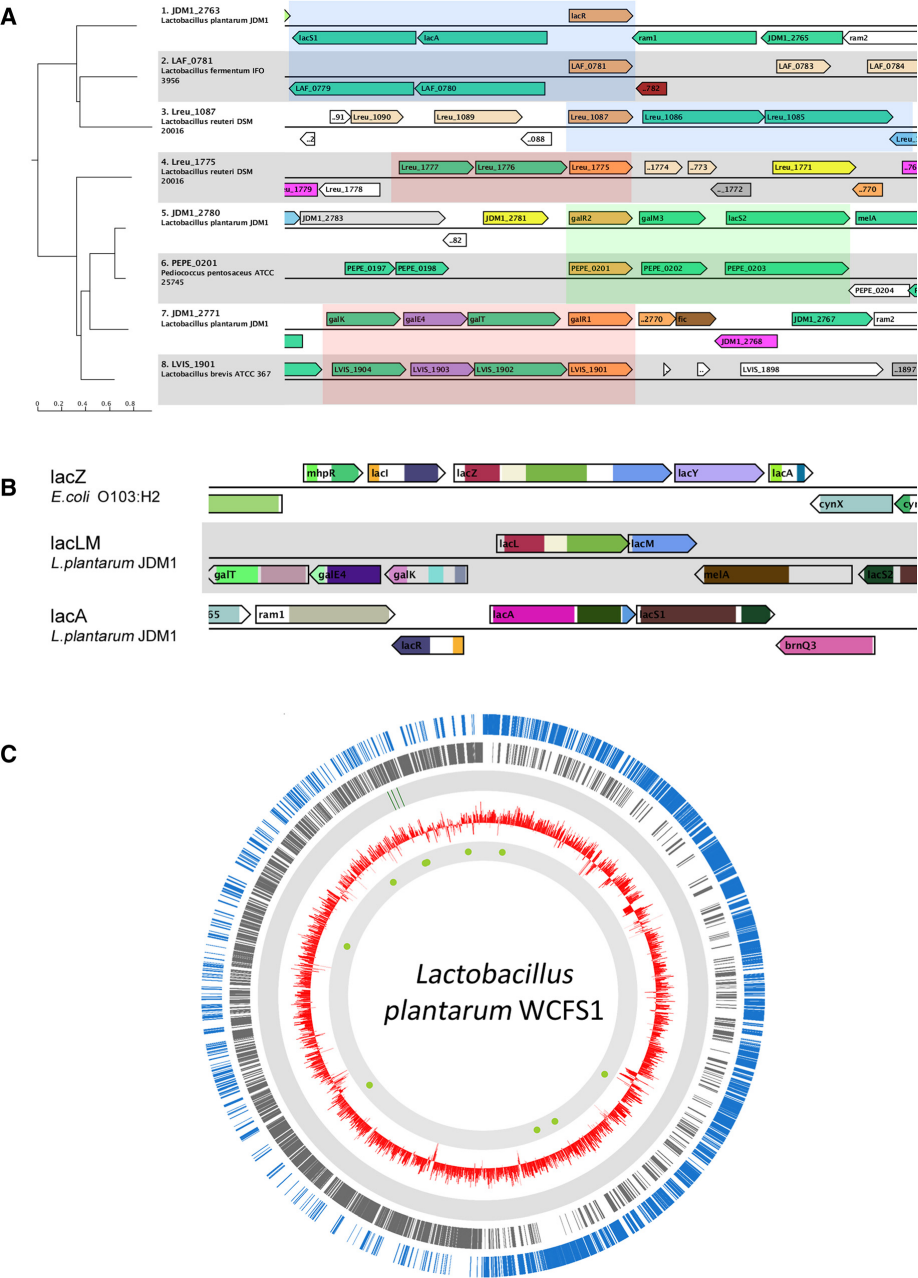
**EbgR-like transcription factors in *L. plantarum *and other lactobacilli. A**) MGcV visualization of a phylogenetic tree of EbgR-type regulators in some Lactobacilli. To simplify, the tree was pruned (species: *Lactobacillus plantarum JDM1* and *WCFS1*, *Lactobacillus fermentum IFO3956*, *Lactobacillus reuteri DSM20016*, *Lactobacillus brevis ATCC 367* and *Pediococcus pentosaceus*). The context range was set to 10.000 nucleotides, genes were colored by COG-class and trivial names were used to label genes. The visual combination of the phylogenetic tree and genomic context allows to distinguish three groups; lacR-, rafR- and galR-like sequences (designated in blue, green and red, respectively). **B**) Comparative context map of the beta-galactosidase encoding genes *lacZ* (*E. coli*), *lacLM* (*L. plantarum*) and *lacA* (*L. plantarum*). Pfam domains are used for gene-coloring and trivial names are used to label genes. In *L. plantarum lacLM* and *lacA* are both annotated to encode a protein with the same name (beta-galactosidase). Yet, from this comparative view it becomes clear that *lacLM* and *lacA* must have a different evolutionary origin. Although lacLM is encoded by two genes, the domain structure appears identical to the single *lacZ* gene of *E. coli*. **C**) A circular genome map of *L. plantarum* in which the ORFs on the plus strand (blue), on the minus stand (grey), the locations of regulator encoding genes *lacR*, *rafR* and *galR* (green), the GC% (red) and putative binding sites (similarity to motif >90% [8]; represented by the green dots) are included.

The study of the *lac* operon and its control in *Escherichia coli* has set the paradigm of bacterial transcription regulation. The associated regulator in *E. coli* was named LacI. Most bacteria contain multiple homologs of this transcription factor family. In lactic acid bacteria, the *lac* operon is often associated with LacI-family regulators that form a separate clade within the family (e.g. EbgR in *E. coli*) [8]. *L. plantarum* WCFS1 has three regulators that belong to this clade: LacR (ortholog in *B. subtilis*[51]), GalR (ortholog in *S. thermophilus*[52]) and RafR [53]. To find functional equivalents in other Lactobacilli, the protein sequences of homologs were collected using a BLAST search. The recovered protein sequences were aligned and a neighbor-joining tree was constructed. To determine the degree of conservation the tree was used as input for MGcV. As shown in Figure 2A, integration with gene-context enhances both the interpretation of the tree and the identification of orthologs. Based on the integrated visualization of the phylogenetic tree and genomic context we can easily distinguish three different clusters. One of the first things that can be done on basis of the integrated view is a specification of annotation information as present in the NCBI database for orthologous genes that share context. The genes *JDM1_2771* (*galR1*) and *JDM_2780* (*galR2*) can be easily re-annotated to *galR* and *rafR*, respectively, on basis of the specific annotation that is available for *L. plantarum WCFS1*. Also the functional equivalency between genes can be evaluated, like for the gene *Lreu_1775*, which is the only regulator of e*bgR*-type in the *Lactobacillus reuterii* genome. Based on the tree and the fact that the gene has a similar gene context as *JDM1_2771* (*galR*) and *LVIS_1901* and not as *JDM_2780* (*rafR*) and *PEPE_0201,* it can be annotated as *galR* with the expected inducer galactose.

The production of galacto-oligosaccharides using microbial beta-galactosidases is currently well-studied in the field of functional foods [54]. In *Escherichia coli* a gene encoding beta-galactosidase: *lacZ*, was described first by Joshua Lederberg in 1948 [55]. It took 25 years before a second beta-galactosidase encoding gene was described [56], which was designated *ebgA* from evolved beta-galactosidase. The discovery resulted in the classic study (designation by [57]) of molecular evolution (review in [58]). The Pfam and COG classification (Figure 2B) comply with the assertion that both genes have evolved from a common ancestor. In many lactobacilli a third closely-related variant is found, *lacLM*. In some Lactobacilli (e.g. *L. delbrueckii* and *L. salivarius*) the protein is encoded by a single gene. However, in most Lactobacilli the protein is encoded by two neighboring genes (probably the result of gene fission) and the active protein is a heterodimer [59]. It is the LacLM protein that is mostly exploited in biotechnological applications [60,61]. Like *E. coli*, various Lactobacilli have a second beta-galactosidase encoding gene, *lacA*. However, this gene has a completely different evolutionary origin and thus represents a functional analog. This conclusion can also easily be derived from the (pfam-) annotation information that is available in MGcV (Figure 2B).

We have maintained the circular viewer of the original MGV in which we constructed a circular genome map of *L. plantarum* (Figure 2C). In this map we included the locations of regulator-encoding genes *lacR*, *rafR* and *galR*, the GC-percentage and putative binding sites (similarity to motif >90% [8]). The genomic segment containing *lacR, rafR* and *galR* is flanking a region with a decreased GC-percentage, which was suggested to represent a lifestyle adaptation region in which many genes are acquired by horizontal gene transfer [62].

#### Case study 2: Reconstruction of GlnR-mediated regulation in Streptococcus mutans

**Figure 3:**
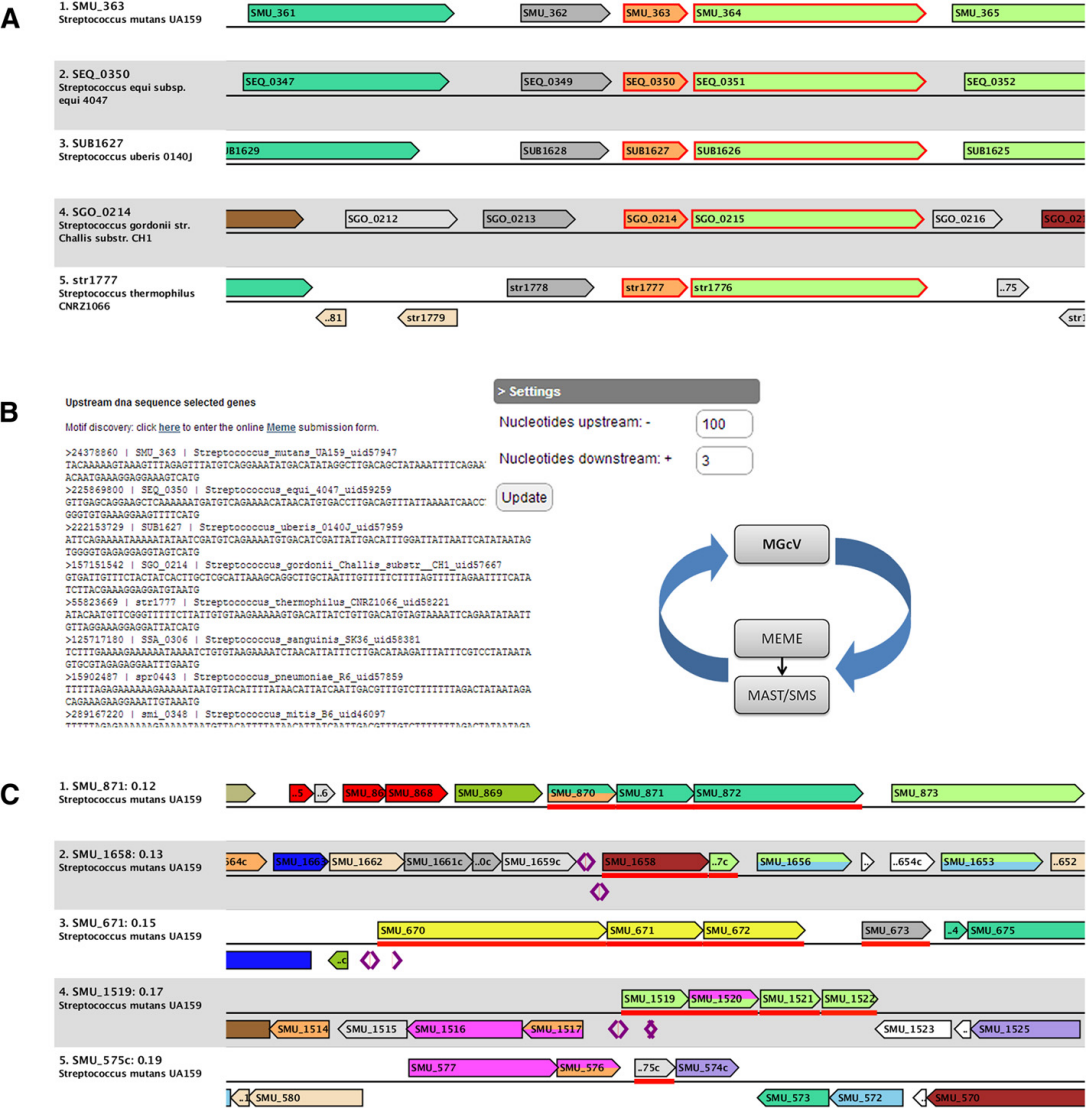
**Application of MGcV in the reconstruction of GlnR-mediated regulation in *Streptococcus mutans*. A**) Comparative context map of GlnR homologs obtained via a BLAST search in all sequenced Streptococci (only five species are shown). The GlnRA operon and its direct genomic context is clearly conserved in the Streptococci. The map was used to graphically select those genes that could be preceded by a binding site. **B**) The “upstream region” option of the “Data-export”-box was used to obtain upstream regions of the selected genes. Subsequently, the available link to MEME was used to search for possible overrepresented motifs. The results were refined and a motif defined [63], which was then used to search and score putative binding sites (e.g. using MAST or SMS). **C**) A comparative context map ranked on expression ratios (low-to-high) of a GlnR mutant, visualized in conjunction with predicted GlnR binding sites. To exemplify, the figure is limited to the top 5 of down-regulated genes. In this map, gene expression ratios are represented in a colored bar (red-to-green gradient; red is down-regulated) at the baseline and putative binding sites are designated by purple arrows (direction representing the strand). Both the microarray data and putative binding sites and their corresponding binding sites were uploaded using the “Data import”-box. The resulting map allows the analysis of the putative GlnR binding sites in light of the expression data of the GlnR mutant. Most of the top down regulated genes (SMU_1658, SMU_671 and SMU,1519 in Figure 3C) indeed are preceded by a putative binding site.

Recently, we have published a comparative genomics study on the transcription factor GlnR [63]. GlnR is one of the four major transcription factors involved in the control of central nitrogen metabolism in *Bacillus subtilis*. A BLAST search was performed to retrieve GlnR orthologs from all sequenced Streptococcal genomes and the gene context for the resulting list was displayed in MGcV (see Figure 3A). We observed a clear conservation of the *glnRA* operon and its genomic context in all Streptococcaceae. MGcV was then used to collect selected upstream regions (Figure 3B). These were analyzed using MEME (via the available link; [32]) to search for a motif representing the GlnR-binding site. The motif was then refined and used to identify and score putative binding sites on all Streptococcal genomes (via e.g. MAST [32] or a Similar Motif Search [64]). Subsequently, the resulting list of putative sites with their corresponding similarity scores was uploaded to MGcV. The view was ranked according to similarity score and the binding site predictions could be evaluated in light of their position relative to the genes. Then, the consistency of the predictions with microarray data was checked visually in MGcV. Chen and colleagues constructed a GlnR gene knockout in *Streptococcus mutans* for which they performed a microarray experiment [43]. These data were retrieved and uploaded and the view was ranked according to microarray ratios (Figure 3C). The view makes immediately clear that the predicted binding-sites are consistent with the microarray data. In addition, the view shows that the operon showing the strongest response (consisting of SMU_870, SMU_871 and SMU_872) is not preceded by a putative binding-site and therefore probably is regulated indirectly. In fact, this operon encodes a PTS system for which no functional relation with nitrogen is described. Interestingly, many of the high-scoring putative binding sites are followed by a binding site in the N-terminus encoding part of the gene (Figure 3C: SMU_671 and SMU_1519), suggesting that this might be a particularity of the regulatory mechanism. Finally, the interactive map provides a convenient overview to determine a possible score threshold for both the predictions as well as the expression data.

## Conclusion

Gene-context conservation is an important genomic property to exploit in genome analyses. Nine years ago we developed a Microbial Genome Viewer [17] to support our efforts in the gene annotation and metabolic reconstruction of the lactic acid bacterium *Lactobacillus plantarum WCFS1*[65,66]. Over the years we have experienced the need for additional functionality and more flexibility to enhance the work on the curation of function annotation and on the reconstruction of transcription regulatory networks. While maintaining the functionality, we have changed the complete setup and developed a new interface to create an adaptable interactive Microbial Genome context Viewer with high speed and versatile functionality to aid small-scale analyses. Both the input and output options of MGcV provide many practical features. The interactive maps allow users to graphically select sets of genes for data retrieval and subsequent analyses. Moreover, the maps provide a single integrated view of the data. The maps are made available in SVG, PNG and PDF format and are hereby suited to use as illustrations in publications, posters and presentations. The MGcV features that constitute its value to the manual analysis of genome sequence include: i) its light-weight and flexible interface; ii) the possibility to a) select multiple genes in the maps and extract gene-related data for these; and b) extract selected upstream regions to be used for further analysis; iii) the visual integration of a user-defined phylogenetic tree and the related gene context; and iv) the visual integration and ranking of microarray data or regulatory element predictions in the context of gene organization. Regarding the regulatory elements, any list of positions linked to a quantitative score can be uploaded, ranked and viewed. Possible applications of MGcV include: annotation refinement, function prediction on basis of a (phylogenetic) tree and conserved gene context, the sequence-based reconstruction of gene regulatory networks, and microarray/RNA-seq data analysis. We have presented two case studies to illustrate the practical applications of MGcV. Altogether, MGcV provides a flexible platform to exploit publicly available genomic data in small scale genome analysis in a fast and convenient manner.

## Availability and requirements

Project name: MGcV

Project home page: http://mgcv.cmbi.ru.nl

Operating system(s): Platform independent

Programming language: Python/SVG/Javascript

Other requirements: Internet browser supporting SVG (Scalable Vector Graphics)

License: None required.

Any restrictions to use by non-academics: none

## Competing interests

The authors declare that they have no competing interest.

## Authors’ contributions

LO and CF designed and coordinated the project. LO developed the web-application and wrote the manuscript. RK contributed to the development of the web-server scripts. RS and CF revised the manuscript. All authors have read and approved the final manuscript.
